# Supplementary material for: Differential DNA methylation in Pacific oyster reproductive tissue in response to ocean acidification
Source: BMC Genomics. 2022 Aug 4;23:556. doi: 10.1186/s12864-022-08781-5 (PMC9351233; doi:10.1186/s12864-022-08781-5)
Supplement: Supplementary file 3 — Additional file 3: Supplementary Figure 1. Principal components analysis (PCA) of CpG loci. PCA of CpG methylation for loci covered at 5x read depth in all samples, with colors differentiating low and ambient pH conditions and shape indicating maturation stage. The PCA did not demonstrate any clear separation of samples by treatment. Supplementary Figure 2. Matrix of pairwise scatter plots for 5x CpG loci. Data is presented for CpG covered at > 5x across all samples. Pearson correlation coefficients for each pairwise comparison are presented in the upper right boxes. [file 12864_2022_8781_MOESM3_ESM.docx]

## **Supplementary Figures**


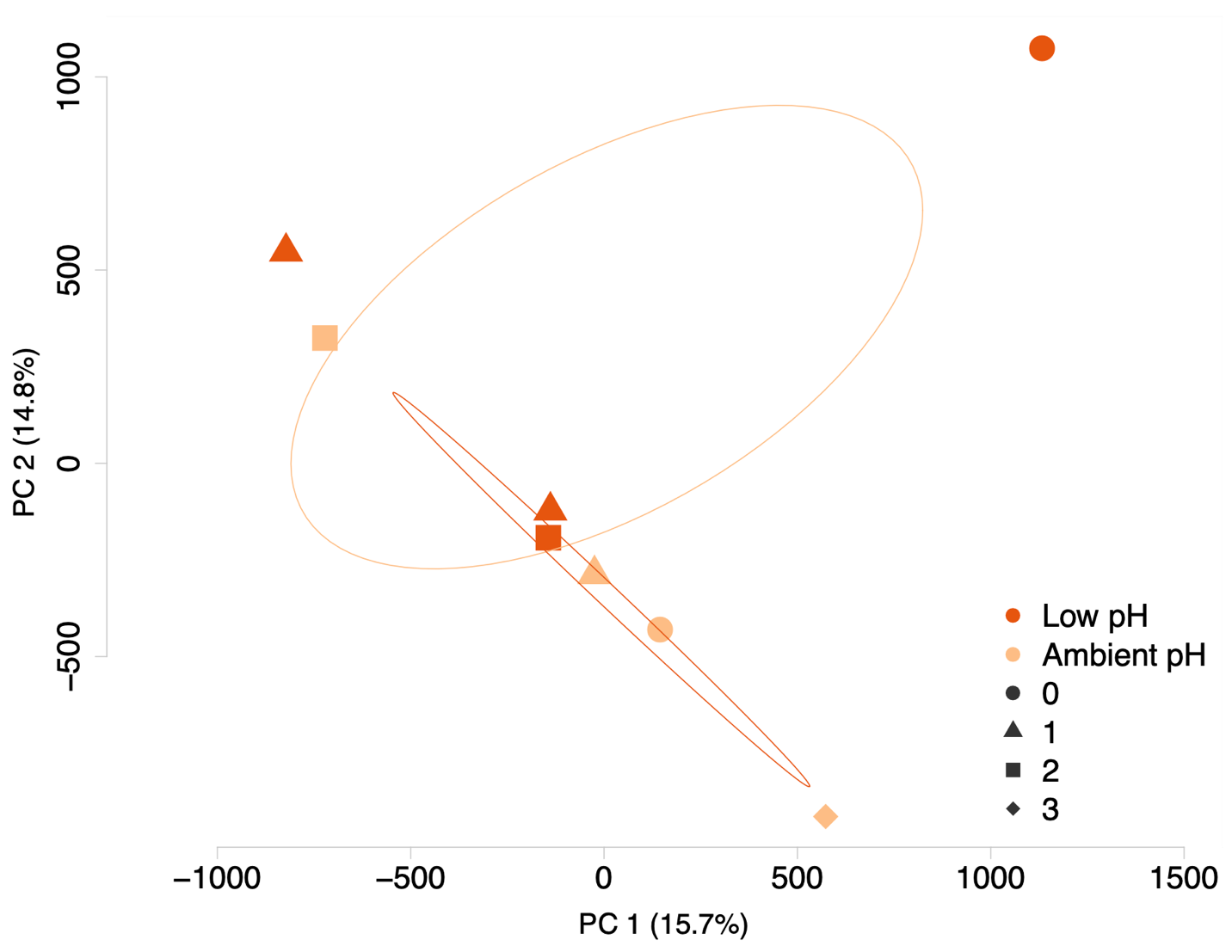


**Supplementary Figure 1. Principal components analysis (PCA) of CpG loci.** PCA of CpG methylation for loci covered at 5x read depth in all samples, with colors differentiating low and ambient pH conditions and shape indicating maturation stage. The PCA did not demonstrate any clear separation of samples by treatment.


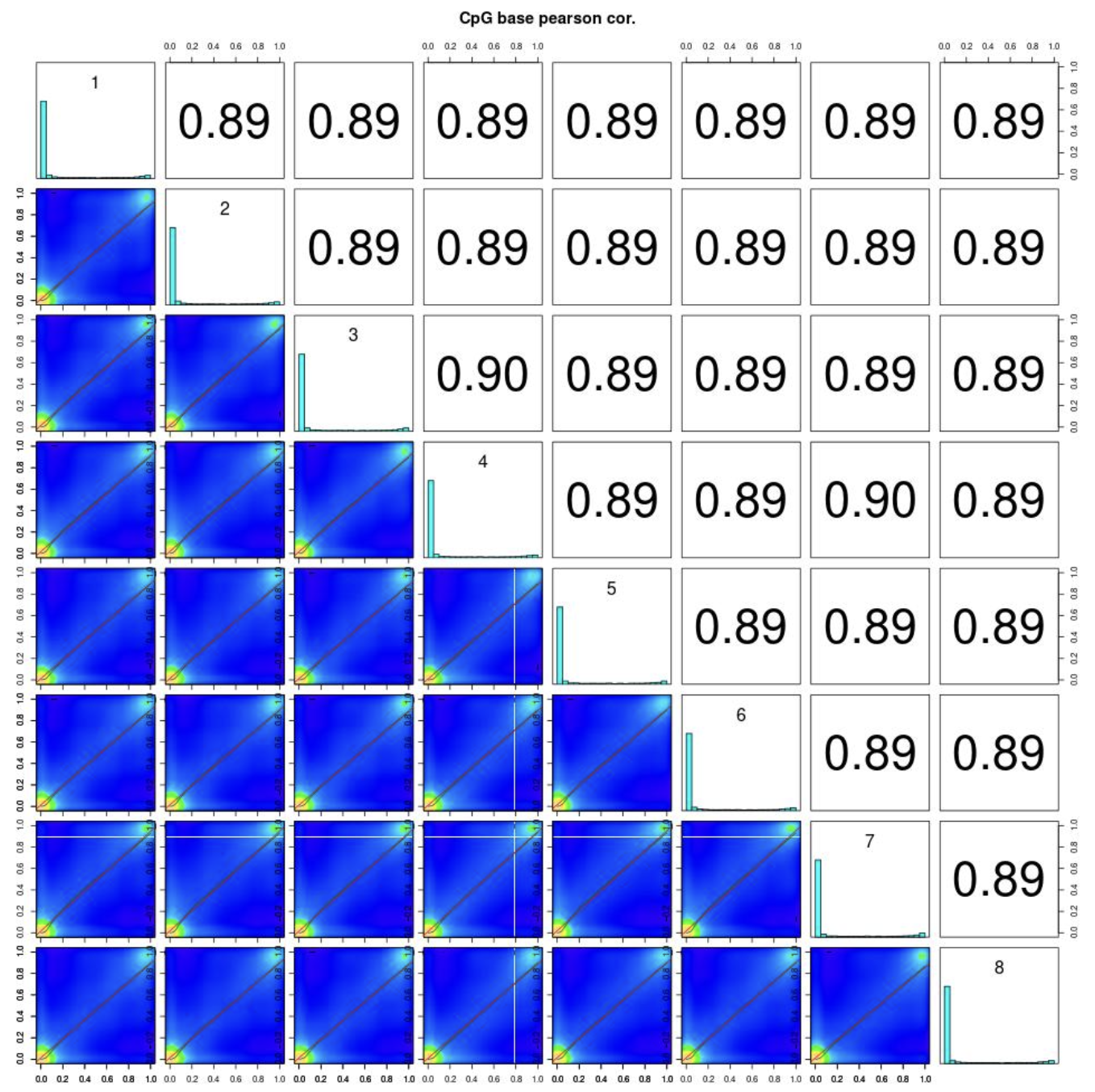
 **Supplementary Figure 2**. **Matrix of pairwise scatter plots for 5x CpG loci.** Data is presented for CpG covered at > 5x across all samples. Pearson correlation coefficients for each pairwise comparison are presented in the upper right boxes.
